# Do We Really Need Omicron Spike-Based Updated COVID-19 Vaccines? Evidence and Pipeline

**DOI:** 10.3390/v14112488

**Published:** 2022-11-10

**Authors:** Daniele Focosi, Fabrizio Maggi

**Affiliations:** 1 North-Western Tuscany Blood Bank, Pisa University Hospital, 56124 Pisa, Italy; 2 National Institute for Infectious Diseases “L. Spallanzani”, 00161 Rome, Italy

**Keywords:** BNT162b2, mRNA-1273, mRNA-1273.211, mRNA-1273.214, mRNA-1273.222, BA.4/5, BA1

## Abstract

The wild-type SARS-CoV-2 Spike-based vaccines authorized so far have reduced COVID-19 severity, but periodic boosts are required to counteract the decline in immunity. An accelerated rate of immune escape to vaccine-elicited immunity has been associated with Spike protein antigenic shifts, as seen in the Omicron variant of concern and its sublineages, demanding the development of Omicron Spike-based vaccines. Herein, we review the evidence in animal models and topline results from ongoing clinical trials with such updated vaccines, discussing the pros and cons for their deployment.

## 1. Introduction

The advent of Omicron sublineages has clearly shown how the efficacy of the currently authorized COVID-19 vaccines (based on the wild-type Spike protein) not only declines in time but can be prone to sudden failure in protecting recipients from severe disease because of major antigenic shifts in Spike proteins. Third-dose boosts have so far counteracted immune escape by inducing heterologous immunity, and the spread of hybrid immunity (vaccination plus convalescence) is further boosting heterologous immunity. Nevertheless, the decline in both the geometric mean titer (GMT) of neutralizing antibody (nAb) in viral neutralization tests (VNT) and the vaccines’ efficacy (VE) at protecting recipients from severe disease restarts after each boost, and the risk of evasive variants is still present. Hence, there are urgent questions regarding whether further boosts should be based on more recent and currently circulating Spike versions, such as BA.1, BA.2, or BA.4/5. Such upgrades can be either a replacement (monovalent vaccine) or a supplement to the backbone of the wild-type vaccine (polyvalent vaccines). The animal models for preclinical vaccine studies in COVID-19 that have been used so far include mice (which, on the contrary of former VOCs, are susceptible to Omicron VOC [1,2,3]) and nonhuman primates. 

Herein, we review the evidence for Omicron-based vaccines (either monovalent or polyvalent) in animal models and early clinical trials, according to the manufacturing platform, and discuss how useful they could be during the next pandemic waves.

## 2. mRNA Lipid Nanoparticle (LNP) Vaccines Based on Spike Receptor-Binding Domain (RBD)

### 2.1. BNT162b2-Omicron

Pfizer (New York, NY, USA)/BioNTech (Mainz, Germany) announced on 15 June 2022 that EMA had initiated a rolling review for the BA.1-adapted vaccine [4]. The review will initially focus on chemistry, manufacturing, and controls (CMC), which relate to the manufacturing of the vaccine [5]. On 25 June 2022, Pfizer announced that the Omicron-adapted monovalent candidate given as a fourth booster dose elicited a 13.5 and 19.6-fold increase in nAb GMT against Omicron BA.1 at 30 µg and 60 µg dose levels; the bivalent vaccine candidate exhibited a 9.1- and 10.9-fold increase against Omicron. The geometric mean ratios for Omicron nAb response are consistent with the regulatory requirement of superiority. Preliminary laboratory studies demonstrated both Omicron-adapted candidates neutralize Omicron BA.4 and BA.5, though to a lesser extent than for BA.1 [6]. The BA.1-adapted bivalent vaccine was submitted to the EMA on 19 July 2022 [7].

The bivalent vaccine contains 15 µg of mRNA encoding the wild-type Spike-protein of SARS-CoV-2 and 15 µg of mRNA encoding the Spike protein of the Omicron BA.4/BA.5 variants; the request for authorization in subjects aged 12 or older was submitted to the FDA on 22 August [8] and granted on 31 August [9], while the same request was submitted to EMA on 26 August [10] and was granted on 12 September [11]. Authorization for usage in children aged 5 through 11 was submitted to the FDA on 26 September [12] and granted on 12 October [13]. On 13 October, the company reported encouraging increases in BA.4/5 nAb titers at 7 days after the boost [14].

### 2.2. mRNA-1273.529 (mRNA-Omicron)

Ying et al. at Washington University reported that a primary vaccination series with **mRNA-1273.529** in mice potently neutralized B.1.1.529 but showed limited inhibition of historical or other SARS-CoV-2 variants. However, boosting this series with mRNA-1273 or mRNA-1273.529 vaccines increased serum nAb titers and protection against B.1.1.529 infection. Nonetheless, the levels of inhibitory antibodies were higher, and the viral burden and number of cytokines in the lungs were slightly lower in mice given the Omicron-matched mRNA booster [15,16].

Gagne et al. at NIAID studied eight macaques that received mRNA-1273 at weeks 0 and 4. They were boosted at week 41 with mRNA-1273 or **mRNA-1273.529 (sometimes referred as mRNA-Omicron)**. The nAb titers against D614G were 4760 and 270 reciprocal ID_50_ at week 6 (peak) and week 41 (pre-boost), respectively, and 320 and 110 for Omicron. Two weeks after the boost, the titers against D614G and Omicron increased to 5360 and 2980, respectively, for mRNA-1273, and 2670 and 1930 for mRNA-Omicron. Following either boost, 70–80% of Spike-specific B cells were cross-reactive against both WA1 and Omicron. The significant and equivalent control of viral replication in the lower airways was observed following either boost. Therefore, an Omicron boost may not provide greater immunity or protection compared to a boost with the current mRNA-1273 vaccine [17].

### 2.3. mRNA-1273.222

Moderna (Cambridge, MA, USA) had previously developed a bivalent (wild-type + Beta) mRNA vaccine [18]. After the advent of Omicron, it developed two BA.1-based candidates: mRNA-1273.211 and mRNA-1273.214. Chalkias et al. reported interim 28-day results of a phase 2/3 trial for mRNA-1273.214. Groups of 437 and 377 participants received 50 μg of mRNA-1273.214 or mRNA-1273, respectively. The median time between the first and second booster doses of mRNA-1273.214 and mRNA-1273 were similar (136 and 134 days, respectively). In participants with no prior SARS-CoV-2 infection, the observed Omicron nAb GMT after the mRNA-1273.214 and mRNA-1273 booster doses were 2372.4 (2070.6−2718.2) and 1473.5 (1270.8−1708.4), respectively, and the model-based GMT ratio (97.5% confidence interval) was 1.75 (1.49−2.04). All pre-specified non-inferiority (ancestral SARS-CoV-2 with D614G mutation [D614G] GMT ratio; ancestral SARS-CoV-2 [D614G] and omicron seroresponse rates difference) and superiority primary objectives (omicron GMT ratio) for mRNA-1273.214 compared to mRNA-1273 were met. Additionally, mRNA-1273.214 at 50 μg induced a potent nAb response against omicron subvariants BA.4/BA.5 (albeit three times lower than those against BA.1) and higher binding antibody responses against Alpha, Beta, Gamma, Delta, and Omicron variants. The safety and reactogenicity profiles were similar and well-tolerated for both vaccine groups [19]. mRNA-1272.214 was approved in the UK by the MHRA on 15 August 2022 [20], by the EMA CHMP on 1 September [21], and in Japan on 12 September [22]. On 19 October 2022 Moderna reported that after its administration as a fourth booster dose in previously vaccinated and boosted participants, a 50 µg booster dose of mRNA-1273.214 elicited a superior nAb response against BA.1 when compared to a 50 µg booster dose of mRNA-1273 at 90 days in all participants regardless of prior infection, as well superior nAb responses against BA.4/5 and BA.2.75 at 28 days [23]. 

On 23 August 2022 Moderna communicated that the bivalent, BA.4/5-based mRNA-1273.222 vaccine had been submitted to the FDA for emergency use authorization [24], which was granted on 31 August for those aged 18 years or older [25], and on 12 October for those aged 6–17 years [26]. mRNA-1273.222 was approved by the EMA’s conditional marketing authorization for patients aged more than 12 on 28 September [27], received a positive opinion by the EMA CHMP on 19 October 2022 [28], and was approved in Japan on 1 November 2022 [29]. On 24 October 2022, researchers in Boston and New York showed that WA1/2020 and Omicron BA.1, BA.2, and BA.5 nAb titers were comparable at 3–5 weeks following monovalent and bivalent mRNA boosters [30,31]. The group in New York also showed comparable titers against BA.4.6, BA.2.75, and BA.2.75.2 [31]. In the BA.5-containing bivalent booster cohort, the neutralizing activity improved against all the Omicron subvariants compared to the monovalent booster cohort; relative to WA1/2020, Davis-Gardner et al. observed a reduction in neutralization titers of 3.7- and 4-fold against BA.1 and BA.5 (vs. 9-15-fold), respectively, and 11.5-(vs. 28) and 21- (vs. 39) fold against BA.2.75.2 and BQ.1.1, respectively [32].

### 2.4. Other mRNA-LNP Vaccines

Fang et al. at Yale tested the activity of an Omicron-specific LNP mRNA vaccine candidate in animals, both alone and as a heterologous booster to the WT mRNA vaccine. The vaccine elicited strong antibody response in vaccination-naïve mice. The mice that received two-dose WT LNP-mRNA showed a > 40-fold reduction in neutralization potency against Omicron than WT two weeks post-boost, which further reduced to a background level after 3 months. The WT or Omicron LNP-mRNA booster increases the waning antibody response of WT LNP-mRNA-vaccinated mice against Omicron by 40-fold at two weeks post injection. Interestingly, the heterologous Omicron booster elicits nAb titers 10–20-fold higher than the homologous WT booster against the Omicron variant, with comparable titers against the Delta variant. All three types of vaccination, including Omicron alone, WT booster, and Omicron booster, elicit broad-binding antibody responses against SARS-CoV-2 WA-1, Beta and Delta variants, and SARS-CoV [33].

Fang et al. at New Haven University developed a Delta + BA.2 bivalent mRNA vaccine candidate and tested it on animals. In mice pre-immunized with two doses of WT LNP-mRNA, all three monovalent boosters and one bivalent booster elevated Omicron nAb titers to various degrees. The boosting effect of Delta- and BA.2-specific monovalent or bivalent LNP-mRNAs is universally higher than that of WT LNP-mRNA, which modestly increased antibody titers in neutralization assays of Omicron BA.5, BA.2.12.1, and BA.2. The Delta and BA.2 bivalent LNP-mRNA showed better performance regarding titer boosting than either monovalent counterparts, which is especially evident in the neutralization of Omicron BA.4 or BA.5. Interestingly, compared to the nAb titers of BA.2 and BA.2.12.1 pseudovirus, the BA.2 monovalent but not Delta and BA.2 bivalent boosters suffered a significant loss of BA.4/5 nAb titer, indicative of a broader activity of the bivalent booster and strong neutralization evasion of Omicron BA.4 or BA.5 even in the BA.2 mRNA-vaccinated individuals [34].

Zang et al. in Shanghai developed an LNP mRNA vaccine (RBD-O). Two doses efficiently induce nAbs in mice; however, the vaccine-elicited antisera were effective only against the Omicron variant but not against the wildtype and Delta strains, indicating a narrow neutralization spectrum. It is noted that the neutralization profile of the RBD-O mRNA vaccine is opposite to that observed for the mRNA vaccine expressing the wildtype RBD (RBD-WT) [35].

Wu et al. in China developed an mRNA vaccine (S_Omicron_-6P) based on an Omicron-specific Spike sequence stabilized by six proline residues [36] (as opposed to the two residues in the currently authorized mRNA vaccines), providing ambiguity as to which of the two variables caused changes in efficacy. In mice, S_Omicron_-6P shows superior nAb-inducing abilities to a clinically approved inactivated virus vaccine, a clinically approved protein subunit vaccine, and an mRNA vaccine (S_WT_-2P) with the same sequence of BNT162b2. Significantly, S_Omicron_-6P induces a 14.4∼27.7-fold and a 28.3∼50.3-fold increase in nAb against the pseudovirus of Omicron and authentic Omicron compared to S_WT_-2P, respectively. In addition, two doses of S_Omicron_-6P significantly protect Syrian hamsters against a challenge with the SARS-CoV-2 Omicron variant and elicits high titers of nAbs in a dose-dependent manner in macaques [37].

Lee et al. in Taipei developed a panel of mRNA-LNP-based vaccines using the receptor-binding domain (RBD) of Omicron and Delta variants, which are dominant in the current wave of COVID-19. In addition to the Omicron- and Delta-specific vaccines, the panel also included a “hybrid” vaccine that uses the RBD containing all 16 point-mutations shown in the Omicron and Delta RBDs, as well as a bivalent vaccine composed of both Omicron and Delta RBD-LNP in a half-dose. Interestingly, both Omicron-specific and Hybrid RBD-LNP elicited extremely high titers of nAb against Omicron itself, but few to no nAb gainst other SARS-CoV-2 variants. The bivalent RBD-LNP, on the other hand, generated antibodies with broadly neutralizing activity against the wild-type virus and all variants. Surprisingly, a similar degree of cross-protection was also shown by the Delta-specific RBD-LNP [38].

The NIAID-funded COVAIL trial (NCT05289037) is testing combinations of vaccines based on a range of variants, including Omicron (BNT162b2 (B.1.1.529), mRNA-1273.529), Beta (BNT162b2 (B.1.351), CoV2 preS dTM (B.1.351), CoV2 preS dTM/D614+B.1.351, and mRNA-1273.351), Delta (mRNA-1273.617.2), and the original strain; it includes mRNA vaccines manufactured by Moderna and Pfizer–BioNTech, as well as an experimental protein-based booster developed by Sanofi in Paris and GSK in London.

## 3. Replicating Omicron mRNA Vaccines

Hawman et al. at NIAID showed that a replicating RNA vaccine made by HDT Bio in Seattle and expressing the B.1.1.529 Spike was immunogenic in mice and hamsters. Mice previously immunized with A.1-specific vaccines failed to elevate nAb titers against B.1.1.529 following B.1.1.529-targeted boosting, suggesting pre-existing immunity may impact the efficacy of B.1.1.529-targeted boosters. Furthermore, the B.1.1.529-targeted vaccine provides superior protection compared to the ancestral A.1-targeted vaccine in hamsters challenged with the B.1.1.529 VOC after a single dose of each vaccine [39].

## 4. Protein Vaccines

Spike protein-based vaccines have been manufactured so far by many different research groups. 

In a mouse model, Ding et al. in Anhui showed that a monovalent vaccine strategy with an individual Spike trimer could only induce binding and nAb against homologous viruses. However, sequential and bivalent immunization with Delta and Omicron Spike trimers could induce significantly broader neutralizing antibody responses against heterogenous SARS-CoV-2. Interestingly, the Spike homotrimer from the Omicron variant showed superior immunogenicity in inducing antibody response against the currently emerging XE variant [40].

Shi et al. in the USA developed a subunit vaccine against Omicron RBD, constructed by masking this epitope with a glycan probe. In immunized mice, it specifically elicited significantly higher-titer nAb than the prototypic RBD protein against Alpha (B.1.1.7 lineage), Beta (B.1.351 lineage), Gamma (P.1 lineage), and Epsilon (B.1.427 or B.1.429 lineage) variant pseudoviruses containing single or combined mutations in the Spike protein, albeit the nAb titers against some variants were slightly lower than against the original SARS-CoV-2. This vaccine also significantly improved the neutralizing activity of the prototypic RBD against pseudotyped and authentic Delta (B.1.617.2 lineage) and Omicron (B.1.1.529 lineage) variants, although the nAb titers were lower than against the original SARS-CoV-2. In contrast to the prototypic RBD, the mutant RBD completely protected human ACE2-transgenic mice from a lethal challenge with a prototype SARS-CoV-2 strain and a Delta variant without weight loss [41].

Du et al. in Guangzhou developed two recombinant Spike trimer protein vaccines based on divalent D614G and BA.1 or BA.1 only. The sera from BA.1 spike protein-vaccinated mice mainly elicited nAb against BA.1 itself. However, a booster with the BA.1 spike protein or a bivalent vaccine composed of D614G and BA.1 spike protein induced not only a potent nAb response against D614G and BA.1 pseudovirus but also against BA.2, the other four SARS-CoV-2 VOC (Alpha, Beta, Gamma, and Delta), and SARS-CoV-2-related coronaviruses (pangolin CoV GD-1 and bat CoV RsSHC014) [42].

Pang et al. reported that three doses of Omicron-RBD immunization elicit comparable nAb titers to three doses of WT-RBD immunization, but the neutralizing activity was not cross-active. By contrast, two doses of WT-RBD with an Omicron-RBD booster increased the nAb GMT against Omicron by nine-fold. Moreover, an additional boost vaccination with the Omicron-RBD protein could increase the humoral immune response against both WT and Omicron [43].

Ye et al. in Hong Kong tested monovalent and bivalent whole-virion-inactivated vaccines (based on wild-type, Delta, and Theta) and found that some cross-variant protection against Omicron and Alpha variants was demonstrated [44].

Appelberg et al. in Sweden designed a DNA vaccine containing RBD loops from the huCoV-19/WH01, Alpha, and Beta variants, combined with the membrane and nucleoproteins that neutralized the huCoV-19/WH01, Beta, Delta, and Omicron virus in vitro [45].

Hevesi et al. in Austria and Spain developed a baculovirus-manufactured protein vaccine based on a divalent gene construct combining the RBD of the Spike protein and the immunodominant region of the viral nucleocapsid. This fusion protein was produced in either *E. coli* or a recombinant baculovirus system. Subsequently, the fusion protein was mixed with adjuvant and administered to mice in a prime-booster mode. The mice (72%) produced an IgG response against both proteins (titer: 10-4-10-5) 14 days after the first booster injection, which was increased to 100% by a second booster. Comparable IgG responses were detected against the delta, gamma, and omicron variants of the RBD region. Durability testing revealed IgGs beyond 90 days [46]

Abdoli et al. reported the chemical inactivation and purification of Omicron Spike, which was then formulated with an alum adjuvant. A full human dose of BIV1-CovIran Plus was injected intraperitoneally to five female mice and two Guinea pigs for the evaluation of abnormal toxicity reactions and pathologic investigations. For the potency evaluation, four groups of 10 mice received two doses of BIV1-CovIran Plus or phosphate-buffered-saline at 7-day and 14-day intervals. The conventional VNT was conducted on sera acquired from vaccinated mice groups seven days after the second injection. There was no evidence of abnormal clinical symptoms or macroscopic or microscopic tissue alterations among the animal models. In all samples from the study group that received two doses of BIV1-CovIran Plus at 7-day intervals, the sera at ≥1/32 times dilution would neutralize the Omicron variant SARS-CoV-2. Similarly, the sera of all samples from the study group, which received two doses of BIV1-CovIran Plus at 14-day intervals, at ≥1/64 times dilution, would neutralize the Omicron variant SARS-CoV-2. Moreover, six out of ten (60%) of the samples in this group would neutralize the Omicron variant of SARS-CoV-2 at 1/128 times dilution. CPE formation was observed in all samples from the control group, and no neutralizing activity was detected at any sera dilution [47].

Novavax (Gaithersburg, Maryland) is testing their own Omicron-based vaccines [48], but details remain undisclosed so far.

## 5. Adenovirus-Based Omicron Vaccines

Swart et al. developed an Ad26 vector encoding an Omicron (BA.1) spike protein (Ad26.COV2.S.529). Ad26.COV2.S.529 encodes for a prefusion-stabilized spike protein, which is similar to the current COVID-19 vaccine Ad26.COV2.S encoding the Wuhan-Hu-1 spike protein. We verified that the spike expression by Ad26.COV2.S.529 was comparable to Ad26.COV2.S. The immunogenicity of Ad26.COV2.S.529 was then evaluated in naïve mice and SARS-CoV-2 Wuhan-Hu-1 spike pre-immunized hamsters. In the naïve mice, Ad26.COV2.S.529 elicited robust nAb against SARS-CoV-2 Omicron (BA.1) but not to SARS-CoV-2 Delta (B.1.617.2), while the opposite was observed for Ad26.COV2.S. In pre-immune hamsters, the Ad26.COV2.S.529 vaccination resulted in robust increases in nAb titers against both SARS-CoV-2 Omicron (BA.1) and Delta (B.1.617.2), while Ad26.COV2.S vaccination only increased nAb titers against the Delta variant [49].

## 6. Live Attenuated Yellow-Fever Virus 17D (YF17D)-Based Vaccines

Sharma et al. created a revised vaccine candidate that carries an evolved Spike antigen. The general methodology for the design and construction of the first YF17D-based SARS-CoV-2 vaccine candidate (YF-S0) has been described [50]. Several mutations were introduced into the original YF-S0 to generate a second-generation vaccine candidate—YF-S0*. The first series of mutations is based on the spike sequence of the Gamma VOC: L18F, T20N, P26S, D138Y, R190S, K417T, E484K, N501Y, D614G, H655Y, T1027I, and V1176F. A second series of mutations is based on a locked spike variant, stabilizing the protein in a more immunogenic prefusion confirmation: A892P, A942P, and V987P [51]. The vaccination of hamsters with this updated vaccine candidate provides full protection against intranasal challenges with all four VOCs, i.e., Alpha, Beta, Gamma (P.1), and Delta (B.1.617.2), resulting in the complete elimination of infectious virus from the lungs and a marked improvement in lung pathology. The vaccinated hamsters also no longer transmitted the Delta variant to non-vaccinated sentinels. The hamsters immunized with our modified vaccine candidate also mounted marked nAb responses against the recently emerged Omicron (B.1.1.529) variant, whereas the old vaccine employing prototypic Spike failed to induce immunity to this antigenically distant virus [52].

## 7. Newcastle Disease Virus (NDV)-Based Vaccines

Gonzalez-Dominguez et al. in New York had previously developed an NDV vaccine expressing the prefusion-stabilized Spike protein from wild-type SARS-CoV-2, named NDV-HXP-S. The vaccine was updated to express the stabilized Spike protein of the Beta, Gamma, and Delta VOC. The trivalent preparation, composed of the ancestral Wuhan, Beta, and Delta vaccines, substantially increases the levels of protection in mice and of cross-neutralizing antibodies against mismatched, phylogenetically distant variants, including the currently circulating Omicron variant [53].

## 8. Conclusions

At this point, it is not clear whether there is an advantage in switching from the current ancestral-based to variant Spike-based vaccines, nor is it clear how important it is that the immunogen in the variant-modified vaccine is antigenically closely related to the Spike protein of the currently circulating variant. For instance, Sanofi–GSK’s adjuvanted recombinant protein MVB.1.351 vaccine, which is based on the Beta variant, triggered higher nAb responses against all variants, including BA.1, than BNT162b2 [54].

Khoury et al. demonstrated that the relative benefits of a variant-modified vaccine are very dependent on the underlying (pre-booster) population immunity to infection for the currently circulating variant [55]. There are controversial data about the protection conferred by multiple natural exposures. A combination of pre-Omicron and Omicron infection-elicited immunity has been shown to be the most protective against BA.2.75* reinfection [56]; nevertheless, simultaneous exposure to the Delta and BA.1 does not confer an additional immune advantage compared to exposure to BA.1 alone [57]. In a large study in Qatar, a history of primary-series vaccination enhanced immune protection against Omicron reinfection, but a history of booster vaccination compromised protection against Omicron reinfection [58]. When it comes to the very recent BQ.1.1.* sublineage, bivalent vaccines induce nAb titers by about twice the degree compared to the monovalent vaccines [32], but in both cases the titers have been so low that the clinical vaccine’s efficacy remains doubtful [59,60].

The composition of adapted COVID-19 vaccines will ultimately depend on the recommendations of public health authorities and the World Health Organization (WHO) as well as the considerations of regulatory bodies such as the EMA and other members of the International Coalition of Medicines Regulatory Authorities (ICMRA). There is evidence for the rapid waning of protection induced by prior BA.1/BA.2 infection against BA.5 infection (compared to uninfected people, RR= 0.12 at 3 months, and RR = 0.24 at 5 months) [61], which suggests that the most recent Omicron sublineages should be eventually used. Accordingly, on 28 June 2022, FDA Advisory Committee (VRBPAC) review, the decision was made to wait for a BA.5 specific vaccine, and in August 2022 the US administration announced plans to procure and administer millions of doses of the BA.4/5 vaccine from Pfizer and Moderna for Autumn 2022 (relying on the speed of the mRNA vaccine platforms). 

Herein, we have shown that Omicron Spike-based vaccines, despite hopes of superiority, only offer non-inferiority in terms of nAb levels against recent Omicron sublineages (Table 1). At this point, it would seem wiser to facilitate campaigns for second boosts with the ancestral vaccines, given the mounting evidence of benefits in subjects older than 55 [62,63,64]. Further studies are needed to assess the impact of further bivalent boosts that could partially remove the immune imprinting and the durability of response and T-cell immunity elicited against SARS-CoV-2. With these caveats in mind, the choice of whether or not to invest in Omicron-specific vaccines for the next boosts is likely to remain based on safety and economical grounds, with updated vaccines likely to have shorter follow-up data and be more expensive than the ones based on wild-type because of obvious R&D costs, decreased competition, and reduced recipient numbers.

## Figures and Tables

**Table 1:** Summary of in vitro neutralization data of sera from individuals comparing geometric mean titers (GMT) after Omicron-updated vs. wild-type mRNA vaccines as fourth doses after three previous doses of mRNA vaccines (* after 2 previous doses of mRNA vaccines; # range 2–4 previous doses).

In Vitro Neutralization Assay	*n* per Arm	Manufacturer	Median Follow-Up Duration	nAb Titer against BA.4/5 (Bivalent vs. Monovalent)	nAb Titer against BQ.1.1 (Bivalent vs. Monovalent)	Ref.
live virus	12	n.a.	16–42 days	11-fold increase (GMT 576) * 4-fold increase compared to 2 monovalent boosts	6-fold increase (GMT 112; higher in 2 previously infected individuals) * 2-fold increase compared to 2 monovalent boosts	[32]
	29	Pfizer or Moderna	1 month	3-fold increase (GMT 298; 1558 in previously infected)	3-fold increase (GMT 73; 267 in previously infected)	
	40 (18–55) + 40 (older than 55)	Pfizer	1 week	no difference	n.a.	[65]
	38 (18–55) + 36 (older than 55)	Pfizer	1 month	9.5-fold increase (GMT 606) in 18-55 2.9-fold increase (GMT 236) in older than 55 (higher in uninfected)	n.a.	[14]
pseudovirus	21	n.a	3–5 weeks	1.2 fold increase (GMT 1649)	n.a.	[31]
	18 (previously 33% infected)	8 Pfizer 10 Moderna	3 weeks	1.3-fold increase (GMT 3693) ^#^	1.2-fold increase (GMT 406) ^#^	[60]
					n.a.	[30,60]

## Data Availability

This manuscript did not generate novel data.
